# Prostatic Artery Embolization: Influence of Cone-Beam Computed Tomography on Radiation Exposure, Procedure Time, and Contrast Media Use

**DOI:** 10.1007/s00270-021-02787-4

**Published:** 2021-03-03

**Authors:** F. Bürckenmeyer, I. Diamantis, T. Kriechenbauer, T. Lehmann, T. Franiel, A. Malouhi, M. O. Grimm, U. Teichgräber, R. Aschenbach

**Affiliations:** 1 Department for Diagnostic and Interventional Radiology, University Hospital Jena, Am Klinikum 1, 07747 Jena, Germany; 2 Center for Clinical Studies, University Hospital Jena, Jena, Germany; 3 Clinic for Urology, University Hospital Jena, Jena, Germany

**Keywords:** Prostatic artery embolization, Radiation exposure, Cone-beam computed tomography, Dose-area product, Contrast media

## Abstract

**Purpose:**

To evaluate the effect of cone-beam computed tomography (CBCT) on radiation exposure, procedure time, and contrast media (CM) use in prostatic artery embolization (PAE).

**Materials and Methods:**

Seventy-eight patients were enrolled in this retrospective, single-center study. All patients received PAE without (group A; *n* = 39) or with (group B; *n* = 39) CBCT. Total dose-area product (DAP_total_; Gycm^2^), total entrance skin dose (ESD_total_; mGy), and total effective dose (ED_total_; mSv) were primary outcomes. Number of digital subtraction angiography (DSA) series, CM use, fluoroscopy time, and procedure time were secondary outcomes. PAE in group A was performed by a single radiologist with 15 years experience, PAE in group B was conducted by four radiologists with 4 to 6 years experience.

**Results:**

For groups A vs. B, respectively, median (IQR): DAP_total_ 236.94 (186.7) vs. 281.20 (214.47) Gycm^2^(*p* = 0.345); ED_total_ 25.82 (20.35) vs. 39.84 (23.75) mSv (*p* =  < 0.001); ESD_total_ 2833 (2278) vs. 2563 (3040) mGy(*p* = 0.818); number of DSA series 25 (15) vs. 23 (10)(*p* = 0.164); CM use 65 (30) vs. 114 (40) mL(*p* =  < 0.001); fluoroscopy time 23 (20) vs. 28 (25) min(*p* = 0.265), and procedure time 70 (40) vs.120 (40) min(*p* =  < 0.001). Bilateral PAE was achieved in 33/39 (84.6%) group A and 32/39 (82.05%) group B(*p* = 0.761), all other patients received unilateral PAE. There were no significant differences between clinical parameters and origins of the prostatic arteries (PA) (*p* = 0.206–1.00).

**Conclusion:**

Operators with extensive expertise on PAE may not benefit from addition of CBCT to DSA runs, whereas for operators with less expertise, CBCT when used alongside with DSA runs increased the overall radiation exposure.

## Introduction

Prostatic artery embolization (PAE) is a minimally invasive procedure that is a new option for treating patients with lower urinary tract symptoms caused by benign prostatic hyperplasia [1]. PAE has advantages over traditional surgical therapies because it is minimally invasive with high rates of technical success and results in improved urinary flow rates and quality of life [2–8]. However, because of its complexity, the procedure demands a high level of investigator skill and long fluoroscopy times as well as angulated views, which can lead to higher radiation exposure for both staff and patients [1]. The longer fluoroscopy times and more angulated views are often related to determine the origins of the prostatic arteries (PA) and to effectively cannulate or evaluate the distal PA anatomy. One approach to this is the use of intraprocedural cone-beam computed tomography (CBCT). CBCT could replace digital subtraction angiography (DSA) to identify the PA anatomy and to help treatment planning during PAE. On the other hand, CBCT increases the radiation exposure of the patient and requires more contrast media (CM) to visualize the vascular anatomy [9–12]. Although PAE procedures are increasingly performed, limited data are available yet to determine the additional radiation dose related to CBCT in patients. The aim of this study was to compare two approaches to PAE—with and without intraprocedural CBCT.

## Methods

The study protocol was conducted in accordance with the Declaration of Helsinki and approved by the local institutional review board. All patients provided written informed consent before the procedure. All patients referred to PAE between January 2015 and May 2018 were retrospectively included in this single-center observational study. Our study group previously published overall data from this cohort [13]. To give a more detailed analysis how CBCT influence the radiation exposure and CM use, this study is a sub-group analysis comparing patients allocated into two groups, group A (procedure without CBCT) and group B (procedure with CBCT). The decision, if CBCT performed was at the discretion of the interventionalist. All interventions were performed using Artis ZeeGo Q system (Siemens, Erlangen, Germany). Except CBCT, all interventionalists used neutral planes and oblique planes according the anatomical needs. CM injection for DSA was performed by hand; 10 mL/injection via guiding catheter and 1 mL/injection via microcatheter. CBCT was performed using a 7-s rotational scan of 180° with an image acquisition of 60 frames per second. Dataset was transferred to a dedicated workstation (Leonardo, Siemens, Erlangen, Germany) to calculate planar datasets and 3D renderings. As described earlier, CBCT was performed placing the catheter in the distal aorta to evaluate both internal iliac arteries and the origins of the PA. 90 mL of CM/saline mixture (60%/40%: total amount of CM 54 mL) was injected with a delay of 4 s and a flow of 8 mL/sec [5]. No computed tomography angiography (CTA) or magnetic resonance angiography (MRA) was performed prior to embolization. PAE in group A was performed by a radiologist with 15 years of experience in embolization therapy. Patients included in group B were investigated by four radiologists, each with 4 to 6 years of experience in interventional therapies. To reduce the bias in experience and with response to the learning curves according the procedure, we excluded the first 50 patients for all interventionalists based on the results of our former study [13].

### Study Outcome Measurements

For all PAE, entrance skin dose of fluoroscopy (ESD_fluoro_), total entrance skin dose (ESD_total_), entrance skin dose of DSA runs, (ESD_series_) dose-area product of fluoroscopy (DAP_fluoro_), total dose-area product (DAP_total_), dose-area product of DSA runs (DAP_series_), number of DSA runs series, and, if performed, CBCT-specific DAP and ESD (DAP_CBCT_; ESD_CBCT_) were gathered using examination reports from the picture archiving and communication system. Procedure time, CM use, and bilaterality of embolization were obtained from the radiological information system.

DAP_total_ (Gy/cm^2^), ESD_total_ (mGy), and effective dose (ED, [mSv]) were the primary measurement outcomes. To calculate the ED from DAP of DSA series (2D imaging) in PAE, we used a conversion coefficient of 0.109 mSv/Gycm^2^ based on Struelens et al. [14]. To estimate ED from DAP_CBCT_, a conversion coefficient of 0.13 mSv/Gycm^2^ was used [15–17].

Secondary outcomes were the number of DSA series, CM use, fluoroscopy time, and procedure time, as well as ESD_fluoro_, ESD_series_; DAP_series_ and DAP_fluoro_. Procedure time was defined as the “table time” of the patient from start to end of the procedure. To compare both groups, age, body mass index, prostate volume, international prostate symptom score (IPSS), international index of erectile function, international index of erectile function erectile domain, quality of life, and peak urinary flow rate were obtained from patients’ clinical records.

To reflect the complexity of the procedures, angiograms were scored side-by-side according to de Assis et al. [18]. All interventionalists scored the angiograms in consensus.

### Statistical Analysis

Continuous variables are reported as median and interquartile range (IQR) or as means with standard deviations (± SD). Primary outcome parameters were compared using Mann–Whitney U test for unpaired independent datasets. Categorial values were compared by two-sided Fisher’s exact test. To compare the rate of bilaterality, we used cross-table chi-square test. The threshold *p* value for significance was *p* = 0.05. Analyses were performed using SPSS software (version 26, IBM Corp., Armonk, NY, USA).

## Results

Thirty-nine patients were included in each group, and patients’ baseline characteristics are summarized in Table 1. We found no significant difference in clinical parameters between the two groups (*p* = 0.206–0.645). The angiographic characteristics according to the PA origins are summarized in Table 2.

**Table 1:** Patients characteristics (n = 78)

	Group A (*n* = 39)		Group B (*n* = 39)		*p*-value
	Median [IQR]	Mean [ ± SD]	Median [IQR]	Mean [ ± SD]	
Age, years	66.8 [11,35]	67.2 [ ± 7.8]	67.6 [10, 26]	69.1 [ ± 6]	0.303
IPSS	25 [8]	24 [ ± 6]	23 [9]	23 [ ± 7]	0.334
IIEF 5	17 [11]	15.6 [ ± 7.6]	16 [16]	14.8 [ ± 7,7]	0.645
IIEF-EF	21 [12]	19.3 [ ± 9.1]	19 [16]	18.2 [ ± 9,1]	0.552
QoL	5 [2]	4.8 [ ± 1.25]	5 [1]	4.56 [ ± 1,25]	0.283
Peak urinary flow rate	10 [5]	10.1 [ ± 3.8]	9 [6]	9.6 [ ± 4]	0.545
Prostate volume, ml	60 [36]	66.7 [ ± 7.8]	62 [37]	72.2 [ ± 39,6]	0.401
Body mass index, kg/m^2^	26.5 [1, 7]	26.9 [ ± 1.15]	27.2 [1, 5]	27.2 [ ± 0.9]	0.206

IPSS *international prostate symptom score*, IIEF-5 *international index of erectile function*, IIEF-EF *international index of erectile function erectile domain*, QoL *quality of life*

**Table 2:** Angiographic characteristics (n = 78)

	Group A	Group B	*p* value
Number PA origin Type I	50/78 (64.1%)	51/78 (65.38%)	1.000
Number PA origin Type II	13/78 (16.66%)	12/78 (15.38%)	1.000
Number PA origin Type III	3/78 (3.85%)	5/78 (6.41%)	0.719
Number PA origin Type IV	12/78 (15.38%)	10/78 (12.82%)	0.819
Number PA origin Type V	0/78 (0%)	0/78 (0%)	1.000

PA *prostate artery*, origins definded according to Assis et al

Type I: origin from the anterior division of internal iliac artery in a common trunk with the superior vesical artery (SVA)

Type II: origin from anterior division of internal iliac artery, inferior to SVA

Type III: origin from obturator artery

Type IV: origin from internal pudendal artery

Type V: less common origins

For primary outcome measurements, median (IQR) DAP_total_ was 236.94 (186.7) Gycm^2^ and 281.20 (IQR 214.47) Gycm^2^, (*p* = 0.345), resulting in median ED_total_ of 25.82 (IQR 20.35) mSv and 39.84 (23.75) mSv for group A and group B, (*p* =  < 0.001), respectively. Median (IQR) ESD_total_ was 2833 (2278) mGy for group A versus 2563 (3040) mGy for group B (*p* = 0.818). Median (IQR) DAP_CBCT_ and ESD_CBCT_ were 70.76 (13.22) and 232 (43.75) in group B, resulting in an ED_CBCT_ of 9.19 (IQR 1.71) mSv.

For secondary outcome measurements, the median (IQR) number of DSA series was 25 (15) in group A and 23 (10) in group B (*p* = 0.16). The median (IQR) amount of CM for the whole procedure was 65 (30) mL in group A and 114 (40) mL in group B (*p* ≤ 0.001). The median (IQR) fluoroscopy time for group A and B was 23 (20) min and 28 (20) min (*p* = 0.265), respectively, and the median (IQR) procedure time was 70 (40) vs. 120 (40) min, which was significantly different (*p* ≤ 0.001). ESD_flouro_ was 252 (282) mGy for GroupA and 634 (603) mGy for Group B (*p* =  < 0.001), DAP_fluoro_ was 18.88 (24.82) Gycm^2^ for Group A and 28.98 (30.44) Gycm^2^ for Group B (*p* = 0.009). ESD_series_ and DAP_series_ were 2585 (2047) mGy vs. 2245 (2675) mGy for GroupA vs.B (*p* = 0.5) and 226.75 (174.5) Gycm^2^ vs. 243.46 (206.31) Gycm^2^ (*p* = 0.593). Bilateral embolization was achieved in 33/39 (84.6%) patients in group A and in 32/39 (82.05%) patients in group B (*p* = 0.761). All other patients received a unilateral PAE, there were no patients with PAE technical failure. Results are summarized in Table 3.

**Table 3:** Results

	Group A (*n* = 39)			Group B (*n* = 39)			*p* value
	Median[IQR]	Mean [SD] 95% CI	% from total	Median[IQR]	Mean [SD] 95% CI	% from total	
DAP_total_, Gycm^2^	236.94 [186.7]	259.09 [ ± 145.62]		281.20 [214.47]	291.23 [ ± 162.59]		0.345
ED_total_, mSv	25.82 [20.35]	28,24 [± 15.87]		39.84 [23.75]	39.59 [ ± 18.5]		< 0.001*
ESD_total_, mGy	2833 [2278]	3036 [ ± 1889]		2563 [3040]	3243 [ ± 2055]		0.818
DAP_CBCT_, Gycm2	n.a	n.a		70.76 [13, 22]	73.85 [ ± 13.53]		n.a
ESD_CBCT_, mGy	n.a	n.a		232 [43.75]	242.15 [ ± 44.06]		n.a
ED_CBCT_, mSv	n.a	n.a		9.19 [1.71]	9.6 [ ± 1.75]		n.a
% of CBCT from total ED	n.a	n.a		23.06	24.24		n.a
Number DSA series, n	25 [15]	28 [ ± 13]		23 [10]	24 [ ± 10]		0.164
Contrast media, ml	65 [30]	69.2 [ ± 33,2]		114 [40]	122.3 [ ± 25.6]		< 0.001*
Fluoroscopy time, min	23 [20]	27.46 [ ± 14.43]		28 [25]	29.74 [ ± 13.44]		0.265
Procedure time, min	70[40]	75.4 [ ± 28]		120[40]	123 [ ± 33]		< 0.001*
ESD_fluoro_, mGy	252 [282]	337.5[ ± 289]	11.11	634 [603]	780 [ ± 692]	24.04	< 0.001*
ESD_series_, mGy	2585 [2047]	2699.28 [ ± 1735]	88.88	2245 [2675]	2463.59 [ ± 1635]	75.96	0.5
DAP_fluoro_, Gycm^2^	18.88 [24.82]	24.50 [ ± 17.76]	9.46	28.98 [30.44]	42.57 [ ± 42.14]	14.63	0.009*
DAP_series_, mGy	226.75 [174.5]	234.59 [ ± 145.62]	90.54	243.46 [206.31]	248.65 [ ± 132.58]	85.37	0.593
Bilateral PAE, n (%)		33/39 (84.6)			32/39 (82.05)		0.761**

ESD *entrance skin dose*, DSA *digital subtraction angiography*, IQR *inter quartile ratio*, SD *standard deviation*, DAP *dose-area product*, CBCT *cone-beam computed tomography*, ED *effective dose* (conversion factor 2D-DAP to ED: 0.109 mSv/Gycm^2^, DAP_CBCT_ to ED: 0.13 mSv/Gycm^2^), *p value are significant different, **p-value calculated with two-sided Fisher´s exact test, PAE *prostatic artery embolization*, % from total is related to mean values

## Discussion

Various reports recommend CBCT in PAE to define origins of the PA, to save CM, limit radiation exposure to patient and staff, and to avoid nontarget embolization and to achieve bilateral embolization [5, 9–11, 19–21]. CBCT was considered helpful in determination of PA origin and in identification of the artery itself, because this remains one of the most challenging parts of the procedure [5, 21]. Although CBCT is considered helpful in identifying PA origin, the additional radiation dose resulting from extra three-dimensional imaging acquisition during PAE is important [17]. Enderlein et al. reported that the combination of CBCT and DSA results in a reduced need for contrast agent and a shortening of fluoroscopy times [22]. In our study, mean fluoroscopy times were 27.46 min (± 14.43) for group A and 29, 74 min (± 13,44) for group B (*p* = 0.265). Although CBCT was available for group B, the fluoroscopy time has no significant difference. This may reflect the lesser experience of the interventionalists in group B. The more frequent use of magnified and angulated views led to a significantly higher ESD (*p* =  < 0.001) and DAP (*p* = 0.009) for fluoroscopy in Group B, despite similar fluoroscopy times between groups. Further, additional use of CM in group B was mostly due to the CBCT performed in the aorta before PAE. The number of DSA series was almost equal and did not differ significantly between groups. Distribution of PA origins was comparable in both groups with around 65% for Type I which is the most challenging type for cannulation [18]. Group A achieved 84.6% of bilateral PAE compared with 82.05% in group B, *p* = 0.761. Although this difference was not significant, it is a clear indicator that PAE is possible with a high rate of technical success in a DSA only protocol. Median total DAP in our study ranged between 236.94 Gycm^2^ (group A) and 281.20 Gycm^2^ (group B), which is within the range of data published by other authors. Andrade (2017) reported a mean DAP of 450.7 Gycm^2^, and Abt et al. (2018) reported a DAP of 176.5 Gycm^2^ [21, 23]. Mean procedure time in our study ranged from 75, 4 (± 28) min to 123 (± 33) min, which is comparable to the procedure times of 89.4 (± 27) min reported by Schott et al. [20].

However, magnetic resonance angiography (MRA) can be helpful to determine the origins of PA effectively before PAE. Zang et al. reported a sensitivity of 91.5% and a positive predictive value of 100% for MRA compared with DSA, and it resulted in a shorter fluoroscopy time and less radiation exposure [24].

The ED is considered the most appropriate quantity for estimating the stochastic risk of exposure to ionizing radiation. However, the complexity of dose calculation for CBCT complicates the comparability of study results. Unfortunately, a variety of conversion factors are available for CBCT because of the heterogeneity of angiographic systems used for acquisition of CBCT, including variations in rotation angles, kilovoltage, fields of views, doses per frame, and rotational speed of acquisition. We used the conversion factor of 0.13 mSv/Gycm^2^ reported by Suzuki et al., which is the best approximation for the system used in our study [15]. CBCT contributed 23% of median ED_total_ to the procedure in Group B. The median (IQR) ED for CBCT was 9.19 (1.71) mSv, which is lower than values reported by Schott et al. (11.8 mSv), Wang et al. (24 mSv), and Desai et al. (14.6 mSv) [9, 19, 25]. In a recent paper, Zumstein et al. compared the results of 22 studies according to radiation dose for PAE [26]. The overall mean DAP was 181.6 Gycm^2^, resulting in an ED of 28.3 mSv. We found an ED of 25.82 mSv for group A and 39.84 mSv for group B, which is within the range reported in the recent literature. Compared with a standard computed tomography scan of the abdomen and pelvis (ED of approximately 11.4 mSv), the radiation dose for patients undergoing PAE is 2 to 4 times higher [26, 27]. This is comparable to the diagnostic reference level for infrarenal endovascular aortic repair (32 mSv) or trans-arterial chemoembolization of the liver (39 mSv) [27]. The overall risk of radiation-induced cancer death for PAE ranged from 0.107% to 0.123% with the highest specific risk for leukemia. Due to significantly lower cell repopulation rate and the reduced life expectancy, these risks become less significant with increasing age [26].

Our study has several limitations. First, we reported only the experience of a single center. Second, no specific conversion factor for our system and procedure is available in the literature, which may have hindered comparability with other studies. Third, the study may have an inherent bias due to the fact that PAE in group A was performed by a single interventionalist with 15 years of experience in embolization therapy, whereas PAE in group B was performed by four interventionalists with experience levels ranging from 4 to 6 years. The learning curve of this procedure affects performance, although we tried to overcome this by excluding data from the first 50 patients in our study, reflecting the results published earlier by our group. [13].

In conclusion, operators with extensive expertise on PAE may not benefit from addition of CBCT to DSA runs, whereas for operators with less expertise, CBCT when used alongside with DSA runs increased the overall radiation exposure. CBCT accounted for up to 23% of the total radiation exposure and raised the CM use of the procedure and should be considered very carefully.
